# Supplementary material for: Upregulated energy metabolism in the Drosophila mushroom body is the trigger for long-term memory
Source: Nat Commun. 2017 Jun 5;8:15510. doi: 10.1038/ncomms15510 (PMC5465319; doi:10.1038/ncomms15510)
Supplement: Supplementary Information — Supplementary Figures and Supplementary Table. [file ncomms15510-s1.pdf]

Supplementary Figure 1

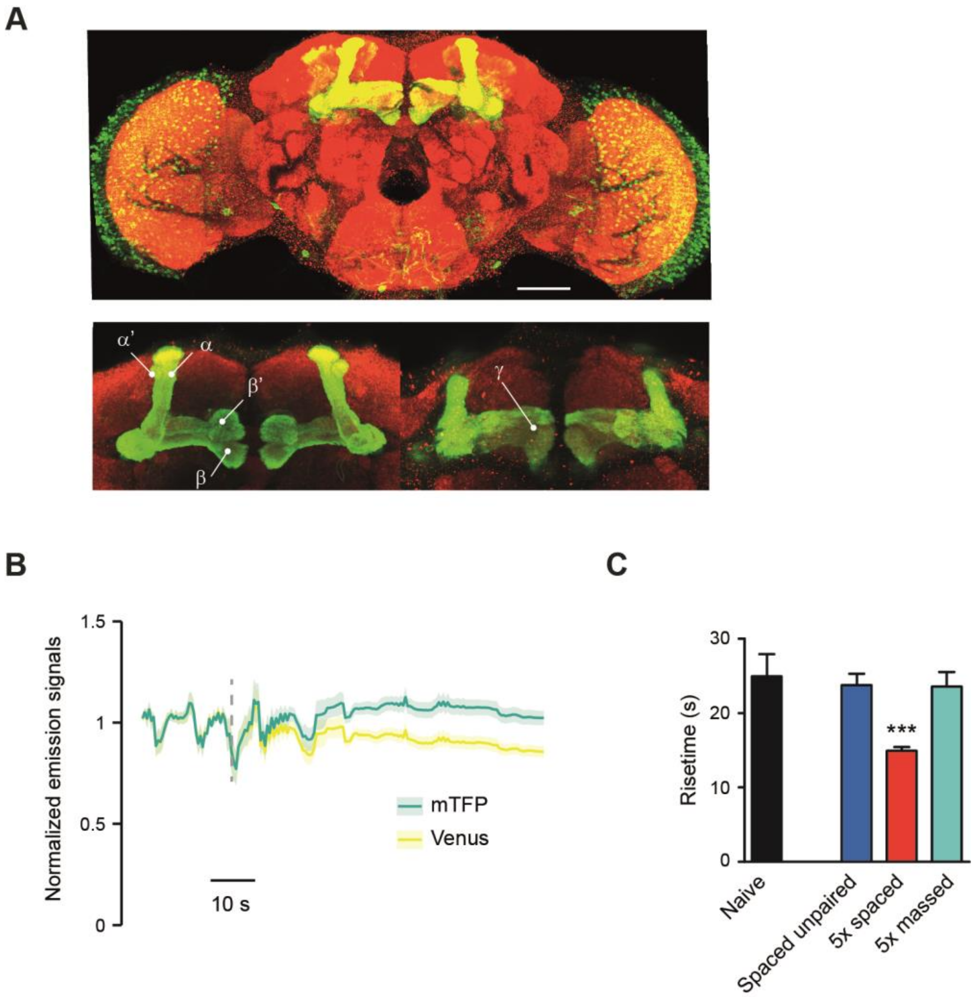

Further characterization of the VT30559 GAL4 line and of the Pyronic sensor

A: Maximum projection of a confocal stack (20x magnification) showing the expression pattern of the VT30559 GAL4 driver, visualized by expression of UAS-mCD8::GFP and anti-GFP labeling (green). The whole brain neuropil was counterstained with anti-nc82 labeling (red). This driver labels essentially MB neurons and neurons in the optic lobes (scale bar: 50µm). The bottom panels are maximum projections of substacks taken at 40x magnification, which show that α/β neurons (forming the α and β lobes), α’/β’ neurons (forming the α’ and β’ lobes) and γ neurons (forming the γ lobes) are labeled. See also Supplementary Movies 1 and 2. **B**: Average time traces of the mTFP and Venus channels recorded in the naive flies shown in Fig. 2A. These traces demonstrate that the increase in the Pyronic ratio indeed originates from a decrease in the FRET between the donor (mTFP) and the acceptor (Venus). **C**: Average risetime from the data shown in Fig. 2A and B. The rise after spaced training was faster than after a spaced unpaired protocol or after massed training ($F_{2,121}=16.97$, $p<0.0001$. Asterisks illustrate the least significant pairwise comparison).

Supplementary Figure 2

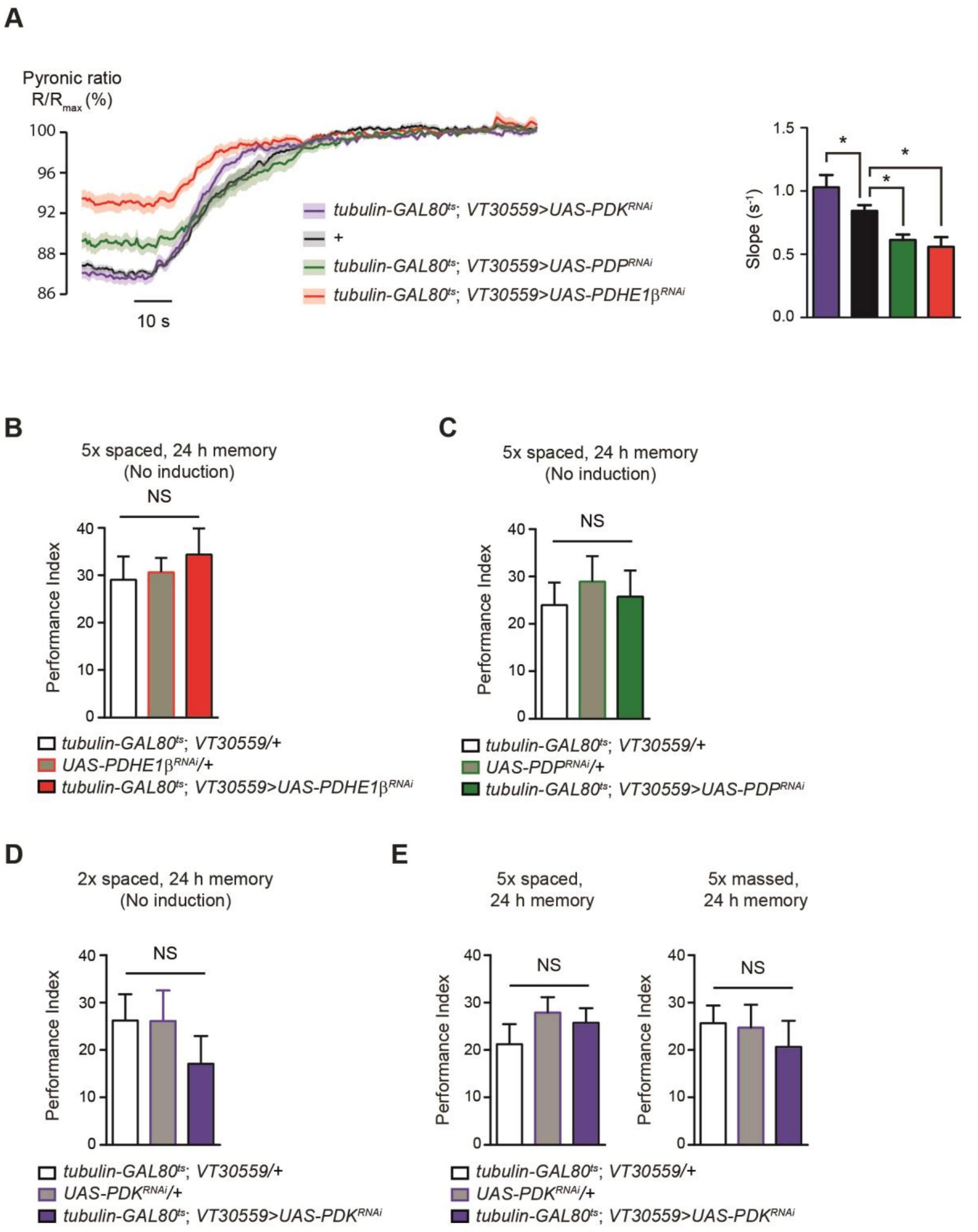

Control experiments for the up- or down-regulation of energy metabolism in MB neurons using RNAi

A: The data presented on Figure 4B were normalized to the plateau value (corresponding to the saturation of the sensor) instead of the baseline before azide application. This presentation highlights the difference in the initial concentration of pyruvate between conditions. With this analysis, slope of pyruvate accumulation were re-calculated. There was still a significant difference between control flies

and each of the three other groups ($F_{3,100}$=9.03, p<0.0001; asterisks illustrate pairwise comparisons). **B**: Without induction of RNAi expression, *tubulin-GAL80^{ts};VT30559>UAS-PDHE1*β^{RNAi} flies showed normal scores as compared to the genotypic controls (n=9, $F_{2,26}$=0.34, p=0.71). **C**: Without induction of RNAi expression, *tubulin-GAL80^{ts};VT30559>UAS-PDP*^{RNAi} flies showed normal LTM scores as compared to the genotypic controls (n=9–11, $F_{2,28}$=0.21, p=0.81). **D**: Without induction of RNAi expression, *tubulin-GAL80^{ts};VT30559>UAS-PDK*^{RNAi} flies did not show increased memory following 2x spaced training (n=9–10, $F_{2,27}$=0.76, p=0.48). **E**: Inhibition of PDK in adult MB neurons had no effect on 24-h memory after 5x spaced training (n=14–15; $F_{2,43}$=0.90, p=0.41), or after 5x massed training (n=12–17; $F_{2,42}$=0.31, p=0.73). NS: not significant in pairwise comparisons.

Supplementary Figure 3

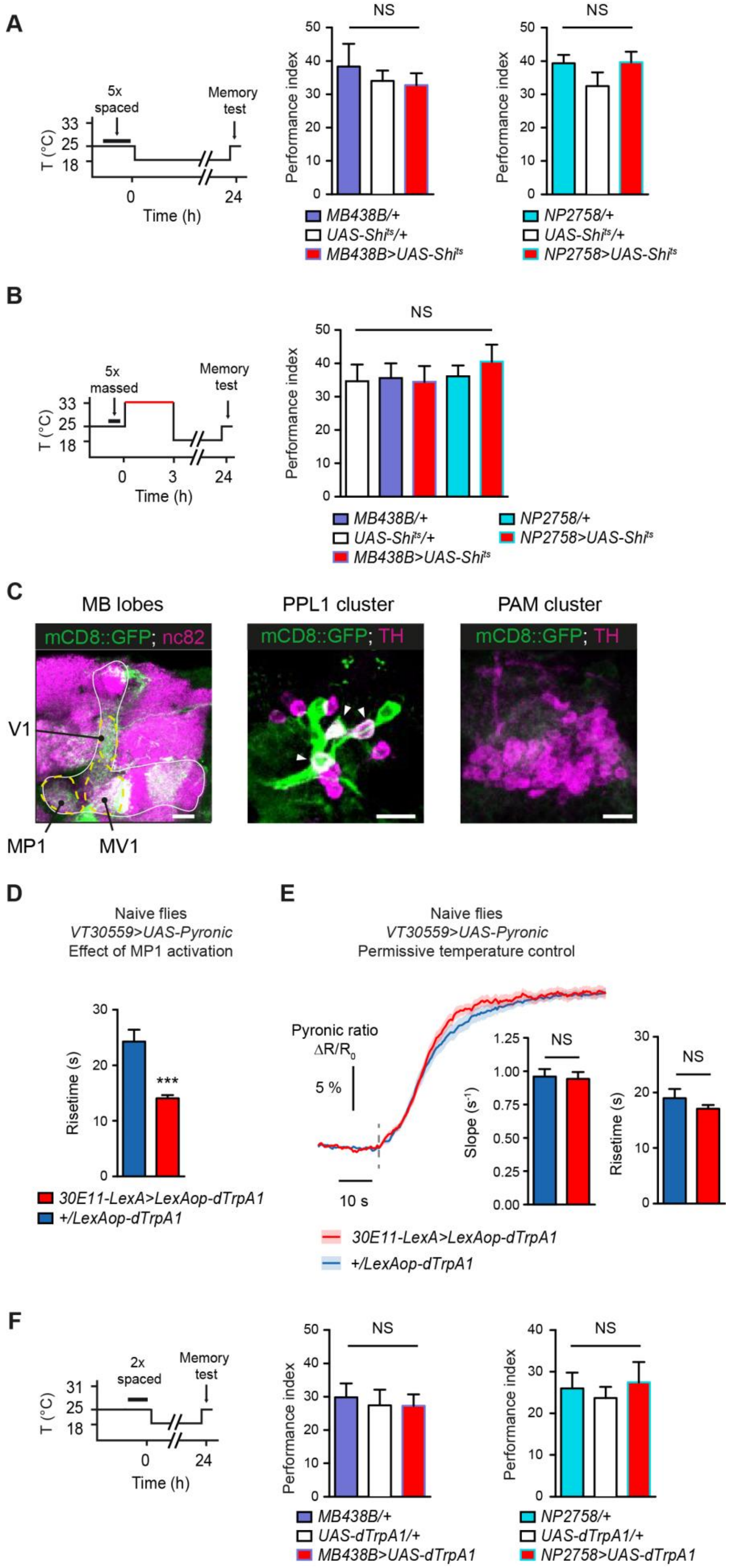

Characterization of the 30E11-LexA line and control experiments for the manipulation of dopaminergic neurons' activity

A: When kept at the permissive temperature after spaced training, flies expressing Shi^{ts} through either the *MB438B* or *NP2758* driver showed normal memory scores as compared to their respective genotypic controls (*MB438B*: *n*=7–8, $F_{2,21}$=0.37, *p*=0.70; *NP2758*: *n*=8–11, $F_{2,26}$=1.43, *p*=0.26). **B**: Flies were submitted to a 3 h period at 33°C following massed training to block the output of Shi^{ts}-expressing neurons. Flies expressing Shi^{ts} through either the *MB438B* or *NP2758* driver had normal 24 h-memory scores as compared to their respective genotypic controls (*n*=9, $F_{4,44}$=0.30, *p*=0.88). **C**: Left panel: expression of mCD8::GFP driven by the 30E11-LexA driver in the MB (white outline). Anti-nc82 labeling was used as a neuropil counterstain, and anti-TH as a marker of dopaminergic neurons. Labeled zones on MB lobes correspond to the projections of MP1, MV1 and V1 neurons (yellow dashed lines), three dopaminergic neurons that belong to the PPL1 cluster. Correspondingly, three cell bodies were co-stained with anti-GFP and anti-TH in the PPL1 cluster (middle panel). An additional zone was labeled on the MB γ horizontal lobes. The only dopamine neurons in the brain that project on to this area are located in the PAM cluster (22). *30E11-LexA* did not label any cell in the PAM cluster (right panel). The three PPL1 neurons are therefore the only dopaminergic neurons labeled by this driver that project on to MB neurons. Scale bars: 10 µm. **D**: The risetime of pyruvate accumulation was lower in flies with activated MP1 neurons (t_{44}=3.94; *p*=0.0003). **E**: In the absence of thermal treatment, no difference was observed between *30E11-LexA>LexAop-dTrpA1* (*n*=17) and *+/LexAop-dTrpA1* flies (*n*=15). The slope and the risetime of pyruvate accumulation were similar between the two conditions (slope: t_{59}=0.21; *p*=0.83; risetime: t_{59}=1.10; *p*=0.27). **F**: In the absence of activation periods, flies expressing dTrpA1 through either the *MB438B* or *NP2758* driver showed similar 24 h-memory after 2x spaced training to that of their genotypic controls (*MB438B*: *n*=8–10, $F_{2,26}$=0.12, *p*=0.89; *NP2758*: *n*=8, $F_{2,23}$=0.24, *p*=0.79). NS: not significant in pairwise comparisons.

Supplementary Figure 4

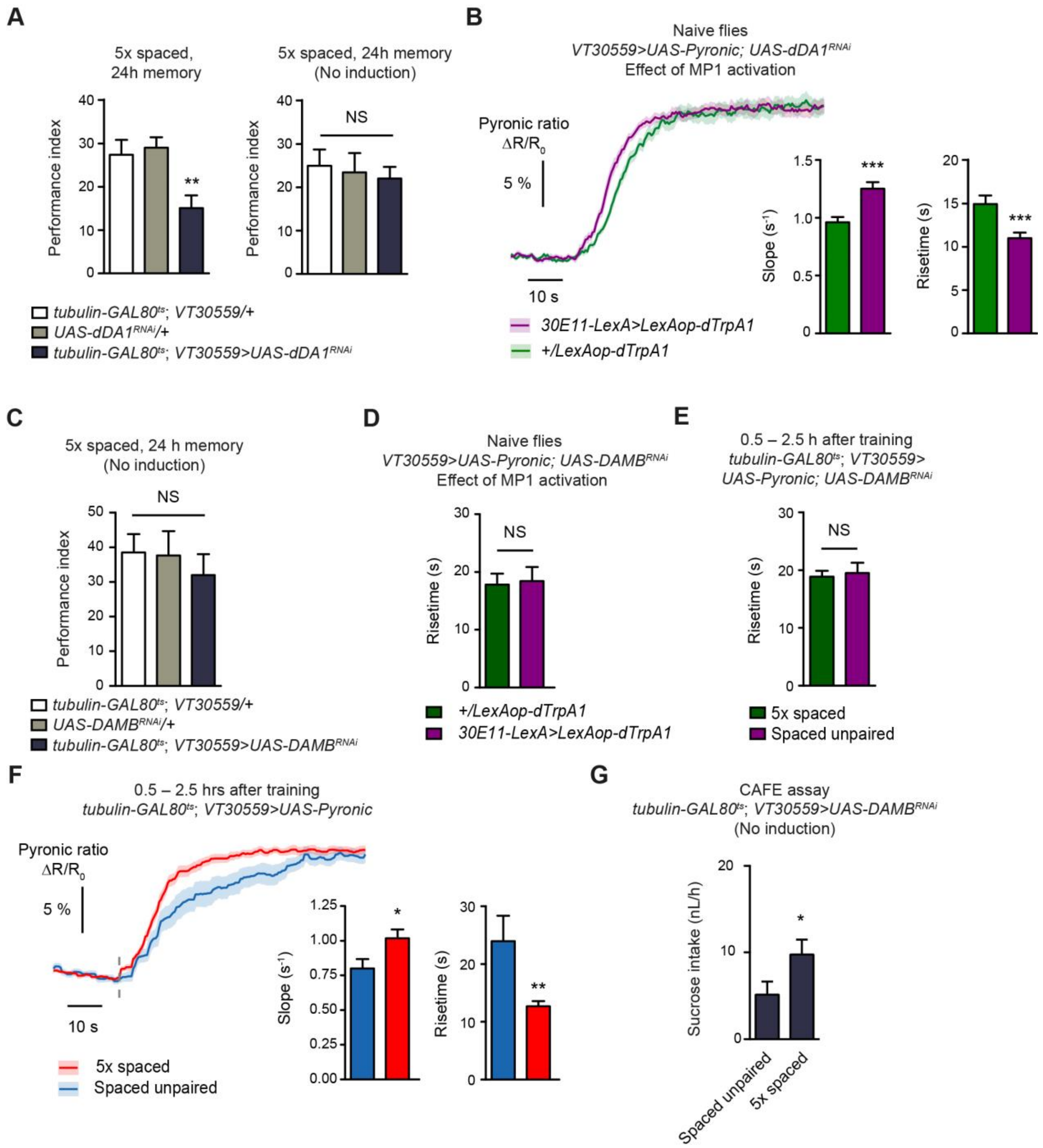

Specificity of the DAMB receptor in the regulation of MB energy metabolism

A: Flies expressing an RNAi construct against dDA1 in MB neurons exclusively at the adult stage showed an LTM defect as compared to their genotypic controls (*n*=17–18; $F_{2,48}$=6.2, *p*=0.004). Without induction of the RNAi, there were no difference between the three groups (*n*=11; $F_{2,32}$=0.16, *p*=0.86). **B**: The RNAi against dDA1 was expressed in MB neurons. In this context, thermal treatment still activated MB energy metabolism in *30E11-LexA>LexAop-dTrpA1* flies (*n*=13) as compared to *+/LexAop-dTrpA1* flies (*n*=11). The slope and risetime of pyruvate accumulation were significantly

different between the two conditions (slope: t_{45}=3.8; *p*=0.0004; risetime: t_{45}=3.5; *p*=0.0009). **C**: Without the induction of RNAi expression, *tubulin-GAL80^{ts};VT30559>UAS-DAMB^{RNAi}* flies showed normal LTM scores as compared to the genotypic controls (*n*=12, $F_{2,35}$=0.33, *p*=0.72). **D**: No difference was measured in the risetime of pyruvate accumulation between *30E11-LexA>LexAop-dTrpA1* and *+/LexAop-dTrpA1* flies (t_{54}=0.21; *p*=0.83). **E**: Risetime of pyruvate accumulation measured in flies shown in Fig. 4C. When RNAi expression was induced, no difference was measured in risetime between the two conditions (t_{34}=0.40; *p*=0.69). **F**: In flies that did not carry the RNAi transgene, but received the thermal induction procedure to induce the expression of the Pyronic sensor, the upregulated energy flux in MB neurons following spaced training could still be observed (5x spaced: *n*=14; unpaired: *n*=13; slope: t_{46}=2.34; *p*=0.023; risetime: t_{46}=2.77; *p*=0.0082). **G**: Without induction of RNAi expression, *tubulin-GAL80^{ts};VT30559>UAS-DAMB^{RNAi}* flies displayed increased feeding behavior following spaced training (*n*=127–128; t_{253}=2.02; *p*=0.044). NS: not significant in pairwise comparisons.

Supplementary Table 1

Genotype	Olfactory acuity (Octanol)	Olfactory acuity (Methyl-cyclohexanol)	Electric shock avoidance
tubulin-GAL80^{ts}; VT30559/+	44.0±5.2	52.7±4.3	58.8±3.7
UAS-PDHE1β^{RNAi}/+	37.1±8.4	40.3±9.8	51.6±4.7
tubulin-GAL80^{ts}; VT30559> UAS-PDHE1β^{RNAi}	47.8±10.8 (*p*=0.99)	62.6±7.0 (*p*=0.58)	53.9±7.7 (*p*=0.99)
UAS-PDP^{RNAi}/+	54.9±8.2	47.3±7.1	67.3±8.1
tubulin-GAL80^{ts}; VT30559> UAS-PDP^{RNAi}	60.5±5.9 (*p*=0.85)	62.3±8.7 (*p*=0.96)	61.6±5.0 (*p*=0.96)
UAS-PDK^{RNAi}/+	44.7±3.6	44.1±7.0	56.4±5.0
tubulin-GAL80^{ts}; VT30559> UAS-PDK^{RNAi}	46.3±4.2 (*p*=0.99)	42.7±5.6 (*p*=0.95)	60.0±7.7 (*p*=0.99)
UAS-DAMB^{RNAi}/+	65.0±11.1	50.9±7.2	50.1±7.0
tubulin-GAL80^{ts}; VT30559> UAS-DAMB^{RNAi}	51.9±10.3 (*p*=0.97)	60.2±9.0 (*p*=0.99)	46.9±4.4 (*p*=0.78)
UAS-dDA1^{RNAi}/+	61.5±9.2	68.3±8.3	67.3±4.9
tubulin-GAL80^{ts}; VT30559> UAS-dDA1^{RNAi}	46.7±5.6 (*p*=0.23)	72.2±4.0 (*p*=0.08)	66.3±2.9 (*p*=0.47)
Wild-type	46.9±7.3	59.4±9.3	59.7±6.4
damb	39.2±9.0 (*p*=0.51)	67.9±9.2 (*p*=0.52)	49.0±4.4 (*p*=0.18)

Control experiments for olfactory acuity and electric shock avoidance

Neither the *damb* mutation, nor the expression in MB neurons of the RNAi constructs used in this study had any significant effect on olfactory acuity, or the avoidance of electric shocks. The *p*-value indicated for *damb* flies is that of a t-test compared to wild-type flies. The *p*-value indicated for RNAi-expressing flies is the lowest one obtained from the two pairwise comparisons between these flies and

their driver (*tubulin-GAL80*^{ts}*; VT30559/+*) or effector (*UAS-...*^{RNAi}*/+*) controls. *n*>20 for *tubulin-GAL80*^{ts}*; VT30559/+* flies; *n*=8–12 for all other groups except *tubulin-GAL80*^{ts}*; VT30559> UAS-dDA1*^{RNAi} (*n*=6 for olfactory acuity and *n*=7 for shock avoidance).
